# Practice effect and test-retest reliability of the Mini-Mental State Examination-2 in people with dementia

**DOI:** 10.1186/s12877-021-02732-7

**Published:** 2022-01-21

**Authors:** Ya-Chen Lee, Shu-Chun Lee, En-Chi Chiu

**Affiliations:** 1 Department of Occupational Therapy, College of Medical and Health Sciences, Asia University, Taichung, Taiwan; 2 Department of Occupational Therapy, Taipei City Psychiatric Center, Taipei City Hospital, Taipei, Taiwan; 3 University of Taipei, Taipei, Taiwan; 4 Department of Occupational Thearpy, I-Shou University, Kaohsiung, Taiwan; 5 Department of Long-Term Care, National Taipei University of Nursing and Health Sciences, No.365, Ming-Te Road, Peitou District, Taipei, 112303 Taiwan

**Keywords:** Mini-Mental State Examination, Practice effect, Reliability, People with dementia

## Abstract

**Background:**

The Mini-Mental State Examination-Second Edition (MMSE-2) consists of three visions: a brief version (MMSE-2:BV), a standard version (MMSE-2:SV), and an expanded version (MMSE-2: EV). Each version was equipped with alternate forms (blue and red). There was a lack of evidence on the practice effect and test-retest reliability of the three versions of the MMSE-2, limiting its utility in both clinical and research settings. The purpose of this study was to examine the practice effect and test-retest reliability of the MMSE-2 in people with dementia.

**Methods:**

One hundred and twenty participants were enrolled, of which 60 were administered with the blue form twice (i.e., the same-form group, [SF group]) and 60 were administered with the blue form first and then the red form (alternate-form group, [AF group]). The practice effect was evaluated using a paired *t*-test and Cohen’s *d*. The test-retest reliability was examined using the intraclass correlation coefficient (ICC).

**Results:**

For the practice effects, in the SF group, no statistically significant differences were found for the MMSE-2:BV and MMSE-2: EV total scores and eight subtests (*p* = 0.061–1.000), except for the MMSE-2:SV total score (*p* = 0.029). In the AF group, no statistically significant differences were found for all three versions of the total scores and subtests (*p* = 0.106–1.000), except for the visual-constructional ability subtest (*p* = 0.010). Cohen’s *d* of all three versions’ total scores and subtests were 0.00–0.20 and 0.00–0.26 for SF group and AF group, respectively. For the test-retest reliability, ICC values for all three versions and eight subtests in SF and AF groups were 0.60–0.93 and 0.56–0.93, respectively.

**Conclusion:**

Our results demonstrated that the practice effect could be minimized when alternate forms of the MMSE-2 were used. The MMSE-2 had good to excellent test-retest reliability, except for three subtests (i.e., visual-constructional ability, registration, and recall). Caution should be taken when interpreting the results of visual-constructional ability, registration, and recall subtests of the MMSE-2.

**Supplementary Information:**

The online version contains supplementary material available at 10.1186/s12877-021-02732-7.

## Background

It has been estimated that about 46.8 million people are suffering from dementia worldwide and that this figure will increase to 74.7 million by 2030 and to 131.5 million people by 2050 [1]. Cognitive function decline is the primary characteristic of dementia that is comprehensively impaired in cognitive function domains such as learning and memory, language, attention, and executive functions [2]. Previous studies had shown that cognitive impairment affects the ability of people with dementia to perform activities of daily living that might have further impact on their and their caretakers’ quality of life [3–5]. Therefore, early detection in order to identify those who are suspected of having cognitive impairment is important.

Four commonly-used cognitive screening tools have been used for the early detection of dementia including the Mini-Mental State Examination (MMSE), the Short Portable Mental Status Questionnaire, the Montreal Cognitive Assessment, and the Saint Louis University Status Examination [6]. Each of abovementioned screening tools has its own merits and among these four, the MMSE has been the most extensively used in clinical and research settings due to its practicality. The MMSE is easy to administer and requires no specialized equipment or training [6–8]. It has been reported that overall, the four screening tools are similar in test-retest reliability; however, the MMSE has demonstrated higher test-retest reliability with acceptable random measurement error and small practice effect [6]. Although the MMSE has its own weaknesses, such as less sensitive to change with increasing age, having a ceiling effect, and vulnerability to practice effect [9, 10], the new version of MMSE, the Mini-Mental State Examination-Second Edition (MMSE-2) was developed to overcome these issues [7].

The MMSE-2 preserves the clinical utility and efficiency of the original MMSE while expanding its application in populations with dementia [7]. The MMSE-2 has two features. First of all, the MMSE-2 is composed of three versions, including a brief version (MMSE-2: BV), standard version (MMSE-2: SV), and expanded version (MMSE-2: EV) (Table 1). Depending on which version is selected, the MMSE-2: BV takes only 5 min while the MMSE-2: SV needs 20 min to complete. The MMSE-2: BV is part of the standard version, which can be used by clinicians to quickly screen patients, and retains the structure and scoring of the MMSE. The MMSE-2: SV is equivalent to the original MMSE [7, 11–13]. The MMSE-2: EV includes two more subtests, story memory and processing speed, to extend the ceiling effect and increase the sensitivity of the MMSE-2 to subcortical vascular dementia [14].

**Table 1:** Overview of administration forms of the MMSE-2

Subtest	Version		
	MMSE-2: BV	MMSE-2: SV	MMSE-2: EV
Registration	V	V	V
Orientation	V	V	V
Recall	V	V	V
Attention and Calculation		V	V
Language		V	V
Visual-constructional Ability		V	V
Story Memory			V
Processing Speed			V

*MMSE2* Mini-Mental State Examination, 2nd Edition, *BV* Brief Version, *SV* Standard Version, *EV* Expanded Version

Secondly, each version of the subtests of the MMSE-2 has two alternate forms (blue and red forms) in order to decrease the practice effects that might occur over repeated testing [7]. Almost all the subtests of the two forms differ in the contents of questions, but the structures are similar (e.g., registration subtest: blue form asks patient to repeat “milk, sensible, before” back to the rater whereas the red form asks “egg, confident, after”. The orientation subtest and attention and calculation subtest remain the same in both forms). The equivalency of the alternate forms of the MMSE-2 has been reported to be 0.96 [7, 12]. These two features have added extra value to the MMSE-2.

An assessment would be considered useful if it could produce stable and consistent results with repeated administration [15]. The practice effect refers to improvements in test results in repeated assessments, given that previous experience might carry over to the next assessment in the absence of any interventions [16]. The practice effect might obscure the true cognitive decline of people with dementia. One of the methods to reduce practice effects is by using alternate forms [17, 18], Test-retest reliability concerns the extent of agreement between repeated assessments under similar assessments conditions [19]. A measure with acceptable test-retest reliability allows users to consistently identify those at risk for cognitive function decline [6]. The abovementioned psychometric properties are essential for a measure to ensure its utility for repeated assessments in people with dementia. Therefore, the purpose of this study was to examine the practice effect and test-retest reliability of the MMSE-2 in people with dementia.

## Methods

### Participants

A convenience sample of people with dementia was recruited from Department of Psychiatry or Department of Neurology of two teaching hospitals in northern Taiwan between March 2019 and April 2020. The following criteria were used to determine the eligibility of people with dementia to participate in this study: (1) diagnosis of probable dementia and dementia according to the National Institute on Aging and Alzheimer’s Association [20]; (2) age ≥ 65 years; and (3) having a stable condition with a stable dose of medication within the past month. The exclusion criteria were: (1) diagnosis of mental retardation, (2) history of severe brain injury, and (3) having different scores on the Clinical Dementia Rating (CDR) over two repeated tests (any change in CDR score was considered an unstable cognitive condition). This study was approved by the Research Ethics Committee of the hospitals. Informed consent was signed by both the participants and their caregivers to confirm their wellness to participate in this study.

### Procedure

Prior to the study, three raters (raters A, B, and C) familiarized themselves with the MMSE-2. The three raters reviewed the user manual of the MMSE-2 and received 4 hours of training from the corresponding author on the administration of the MMSE-2. Then, the three raters performed the MMSE-2 on the corresponding author, and their score results were checked. If there were any discrepancies in the score results, discussions with the corresponding author were required to ensure proper administration procedures and scoring. Finally, the three raters independently administered the MMSE-2 to five people with dementia. The corresponding author observed the assessments and gave MMSE-2 scores simultaneously to confirm that all three raters performed the MMSE-2 correctly in a standardized manner. In addition, when the study began, the raters did not discuss the results of scores with each other to avoid potential bias.

#### The same-form group, SF group

The blue form was administered twice in a two-week interval by raters A and B to the participants, who were from hospital A.

#### The alternate-form group, AF group

The alternate forms (i.e., the blue form at the first assessment and the red form at the second assessment) were administered by rater C 2 weeks apart to the participants who were from hospital B. The AF group completed the MMSE-2 in a fixed order (i.e., the blue form first and red form second).

All assessments were conducted in a quiet room to avoid interference that might have affected the performance of the participants. Between each assessment, the participants were allowed to break to minimize fatigue. The demographic data of all participants were collected from their medical records.

### Measures

The MMSE-2 was developed to assess cognitive impairment. The MMSE-2: BV (score range: 0–16) is composed of three subtests: registration, orientation, and recall. The MMSE-2: SV (score range: 0–30) is composed of six subtests: three subtests of the MMSE-2:BV along with attention and calculation, language, and visual-constructional ability. The MMSE-2: EV (score range: 0–90) is composed of eight subtests: six subtests of the MMSE-2:SV, as well as story memory and processing speed (Table 1) [14]. A higher total score indicates better cognitive function [7].

The CDR measured the level of dementia severity and was used to examine whether the symptom severity of participants was stable during the test and retest sessions. The CDR is composed of six domains, including orientation, memory, judgment and problem solving, community affairs, home and hobbies, and personal care [21]. A global CDR score can be obtained from the six domains to quantify the severity of dementia on a five-point grade scale (0, 0.5, 1, 2, and 3), where 0 indicates no dementia, 0.5 indicates questionable dementia, 1 indicates mild dementia, 2 indicates moderate dementia, and 3 indicates severe dementia [22]. The CDR has been reported to have satisfactory reliability and validity in patients with dementia [23].

### Data analysis

#### Practice effect

Paired *t*-tests were performed to evaluate statistically significant differences between the two assessments. In addition, we calculated Cohen’s *d* as effect sizes to assess the magnitude of change. Effect sizes between 0.00–0.09 were categorized as no practice effect, 0.10–0.19 as trivial, 0.20–0.49 as small, 0.50–0.79 as medium, and ≥ 0.80 as large-sized effects [24]. As multiple *t*-tests were conducted, the significance level was adjusted using a Bonferroni correction [25] for 11 *t*-tests by dividing the significance level of .05 by 11, resulting in a significance level of *p* < .0045. Data were analyzed with IBM SPSS Statistics (Version 22.0; IBM Corp., Armonk, NY).

#### Test-retest reliability

To examine the test-retest reliability of the MMSE-2, the intraclass correlation coefficient (ICC_2,1_) was calculated using a two-way random analysis of variance with absolute agreement. An ICC value of 0.81–1.00 indicates excellent reliability, 0.61–0.80 indicates good reliability, 0.41–0.60 indicates moderate reliability, and < 0.40 indicates poor reliability [26]. In addition, the minimal detectable change (MDC at the 95% confidence level, MDC_95_) was calculated on the basis of the standard error of measurement (SEM) to estimate the random measurement error of the MMSE-2 [27]. In Formula 1, SD represents the standard deviation of all scores of the repeated assessments. In Formula 2, a value of 1.96 was used for the confidence interval of a standard normal distribution (i.e., 1.96 for the 95% confidence level in this study). The $\sqrt{2}$ multiplier represents the additional uncertainty when different scores from the repeated assessments are used.

1 $$\mathrm{SEM}=\mathrm{SD}\sqrt{1-\mathrm{ICC}}$$

2 $$\mathrm{MDC}=1.96\times \sqrt{2}\times \mathrm{SEM}$$

We also calculated the MDC percentage (MDC%), which was independent of the units of measurement, and used it to determine a relatively true change between two assessments: MDC% = (MDC/ highest score of all test data) × 100 [28]. In this study, an MDC% of less than 30% was considered an acceptable random measurement error [29].

## Results

A total of 120 participants participated in this study; of these, 60 from hospital A were assigned to the SF group and 60 from hospital B were assigned to the AF group. The participants in the SF group had a mean age of 81.5 ± 7.8 years, 55% were female, and 58.3% had an educational level below elementary school. The participants in the AF group had a mean age of 79.1 ± 6.8 years, 67.3% were female, and 43.3% had an educational level below elementary school. There were no significant group differences in age, *t* = 1.781, *p* = 0.080, gender, χ^2^ = 0.094, *p* = 0.759, education, *t* = 39.992, *p* = 0.105, or CDR, *t* = 3.436, *p* = 0.752. The characteristics of the participants are shown in Table 2.

**Table 2:** Characteristics of the participants

Characteristic	Same-forms group (*n* = 60)	Alternate-forms group (*n* = 60)	χ^2^or *t*	*p* value
Age (years), mean ± SD	81.45 ± 7.84	79.10 ± 6.75	1.781	0.080
Gender, n (%)			0.094	0.759
Male	27 (45.00)	19 (31.67)		
Female	33 (55.00)	41 (68.33)		
Education, n (%)			39.992	0.105
Below elementary school	35 (58.33)	26 (43.33)		
Junior to senior high school	18 (30.00)	23 (38.33)		
College and above	7 (11.67)	11 (18.33)		
CDR, mean ± SD	1.20 ± 0.56	1.55 ± 0.78	3.436	0.752

*SD* standard deviation, *CDR* Clinical *Dementia* Rating

### Practice effect

In the SF group, no statistically significant differences were found between the two assessments for the MMSE-2:BV and MMSE-2: EV total scores and eight subtests (*p* = 0.061–1.000). There was a statistically significant difference in the MMSE-2:SV total score (*p* = 0.029). The Cohen’s *d* for all three versions total scores and subtests, except for the visual-constructional ability subtest, ranged from 0.00 to 0.15, indicating no or trivial practice effects. Cohen’s *d* for the visual-constructional ability subtest was 0.20, indicating a small practice effect (Table 3).

**Table 3:** Practice effects and test-retest reliability of the MMSE-2 in the same-form group (n = 60)

Versions and subtests	First assessment Mean ± SD	Second assessment Mean ± SD	paired *t*-test (*p* value)	Cohen’s *d*	ICC (95% CI)	MDC (MDC %)
Registration	2.20 ± 1.12	2.28 ± 1.04	−0.71 (0.480)	0.07	0.65 (0.48, 0.78)	0.52 (17.41)
Orientation	3.85 ± 2.63	4.00 ± 2.80	−0.80 (0.429)	0.06	0.86 (0.77, 0.91)	0.52 (5.25)
Recall	0.33 ± 0.71	0.33 ± 0.71	0.00 (1.000)	0.00	0.76 (0.63, 0.85)	0.23 (7.78)
**Brief Version total score**	6.38 ± 3.59	6.62 ± 3.66	−1.08 (0.287)	0.07	0.89 (0.83, 0.93)	0.55 (3.44)
Attention and Calculation	1.58 ± 1.71	1.72 ± 1.69	−0.75 (0.458)	0.08	0.67 (0.50, 0.79)	0.77 (15.49)
Language	5.33 ± 1.61	5.57 ± 1.63	−1.91 (0.061)	0.15	0.82 (0.72, 0.89)	0.40 (5.05)
Visual-constructional ability	0.40 ± 0.49	0.50 ± 0.50	−1.76 (0.083)	0.20	0.60 (0.42, 0.74)	0.28 (27.69)
**Standard Version total score**	13.70 ± 5.74	14.42 ± 5.73	−2.24 (0.029*)	0.13	0.90 (0.84, 0.94)	0.79 (2.74)
Story Memory	3.22 ± 2.60	3.63 ± 2.96	−1.57 (0.123)	0.15	0.72 (0.58, 0.82)	1.08 (9.00)
Processing Speed	3.87 ± 4.07	3.38 ± 3.96	1.69 (0.096)	0.12	0.84 (0.75, 0.90)	0.89 (4.23)
**Expanded Version total score**	20.78 ± 10.53	21.43 ± 10.81	−1.26 (0.214)	0.06	0.93 (0.88, 0.96)	1.03 (2.10)

*MMSE-2* Mini-Mental State Examination, 2nd Edition, *SD* standard deviation, *ICC* intraclass correlation coefficient, *CI* confidence interval, *MDC* minimal detectable change

Significant difference after Bonferroni correction at *p* < .05/11(*p* < .0045)

In the AF group, no statistically significant differences were found between the two assessments of the total scores and subtests (*p* = 0.106–1.000), except for the visual-constructional ability subtest (*p* = 0.010). Cohen’s *d* for all three versions’ total scores and subtests, except for the visual-constructional ability subtest, ranged from 0.00 to 0.10, indicating no to trivial practice effects. Cohen’s *d* for the visual-constructional ability subtest was 0.26, indicating a small practice effect (Table 4).

**Table 4:** Practice effects and test-retest reliability of the MMSE-2 in the alternate-form group (*n* = 60)

Versions and subtests	First assessment Mean ± SD	Second assessment Mean ± SD	paired *t*-test (*p* value)	Cohen’s *d*	ICC (95% CI)	MDC (MDC %)
Registration	2.00 ± 1.25	2.08 ± 1.24	−0.55 (0.583)	0.06	0.56 (0.36, 0.71)	0.76 (25.19)
Orientation	4.50 ± 2.88	4.48 ± 3.06	0.07 (0.946)	0.01	0.80 (0.68, 0.87)	0.82 (8.19)
Recall	0.25 ± 0.63	0.20 ± 0.58	0.69 (0.496)	0.08	0.56 (0.36, 0.71)	0.37 (12.21)
**Brief Version total score**	6.75 ± 4.04	6.77 ± 4.07	−0.05 (0.961)	0.00	0.79 (0.67, 0.87)	1.17 (7.34)
Attention and Calculation	1.95 ± 1.88	1.82 ± 1.83	0.87 (0.387)	0.07	0.80 (0.68, 0.87)	0.51 (10.24)
Language	5.03 ± 2.19	5.03 ± 2.16	0.00 (1.000)	0.00	0.79 (0.67, 0.87)	0.63 (7.77)
Visual-constructional ability	0.42 ± 0.50	0.55 ± 0.50	−2.65 (0.010*)	0.26	0.68 (0.50, 0.80)	0.22 (22.25)
**Standard Version total score**	14.13 ± 7.14	14.18 ± 7.40	−0.11 (0.912)	0.01	0.89 (0.82, 0.93)	1.10 (3.94)
Story Memory	1.77 ± 2.65	1.72 ± 2.45	0.19 (0.853)	0.02	0.67 (0.50, 0.79)	1.16 (9.68)
Processing Speed	3.45 ± 4.14	3.88 ± 4.30	−1.64 (0.106)	0.10	0.88 (0.81, 0.93)	0.70 (4.11)
**Expanded Version total score**	19.35 ± 12.22	19.80 ± 12.29	−0.75 (0.454)	0.04	0.93 (0.89, 0.96)	1.18 (2.57)

*MMSE-2* Mini-Mental State Examination, 2nd Edition, *SD* standard deviation, *ICC* intraclass correlation coefficient, *CI* confidence interval, *MDC* minimal detectable change

Significant difference after Bonferroni correction at *p* < .05/11(*p* < .0045)

### Test-retest reliability

In the SF group, all three versions and subtests, except for the visual-constructional ability subtest (ICC = 0.60), showed good to excellent reliability (ICC = 0.67–0.93) (Table 2). At the 95% confidence level, the MDC values of all three versions and eight subtests ranged from 0.23 to 1.08. The MDC% of all three versions and eight subtests were all < 30% (ranging from 2.10 to 27.69%).

In the AF group, all three versions and subtests, except for the registration and recall subtests (ICC = 0.56), showed good to excellent reliability (ICC = 0.67–0.93) (Table 3). At the 95% confidence level, the MDC values of all three versions and eight subtests ranged from 0.37 to 1.18. The MDC% of all three versions and eight subtests were all < 30% (ranging from 2.57 to 25.19%).

## Discussion

We found that the MMSE-2:SV total score appeared significantly different over repeated assessments in the SF group, which might have led to an examiner’s misinterpretation of a patient’s progress in cognitive function when the same form of the MMSE-2:SV total score was used. The MMSE-2:SV has been reported to be equivalent to the original MMSE [11], which might have increased familiarity in our participants and, as a result, increased the likelihood of practice effects. However, the results showed that when the alternate form of the MMSE-2: SV was used, no significant difference was found between the two assessments. Therefore, based on our results, it is important to use the alternate form of MMSE-:SV when repeatedly assessing cognitive functional status in people with dementia. In addition, we found that Cohen’s *d* values for all three versions and almost all subtests in the AF group were relatively smaller than the values in the SF group. These results demonstrate that the practice effects were mitigated by using alternate forms. Thus, based on our findings, it is beneficial to use the alternate forms of the MMSE-2 in retests in order to minimize practice effects in people with dementia.

The visual-constructional ability subtest in both the SF and AF groups showed small effects. A possible reason for the slightly higher practice effect might be that the same question was used both in the red and blue form of the MMSE-2, with participants being asked to draw two intersecting pentagons in which the interconnected area should be shaped like a rhombus [9, 30]. Our participants might have become familiar with the drawing upon repeated administrations. To reduce the impact of practice effects, there is a need to develop a new question design for the alternate form of the visual-constructional ability subtest.

A measure with sufficient test-retest reliability allows users to obtain stable and consistent results over time when repeatedly used [31]. Our results showed that the three versions of the MMSE-2 total scores (the same or alternate forms) all revealed good to excellent test-retest reliability. These results demonstrate that it is reliable to use the same forms or alternate forms of the MMSE-2 total scores to monitor the changes in cognitive function of people with dementia over time. In addition, because of the good and excellent test-retest reliability found in the three versions of the MMSE-2 total scores in the AF group, these results could imply that the blue and red forms of the MMSE-2 total scores are equivalent to one another in repeated assessments.

The two subtests (i.e., registration and recall) in the AF group showed moderate test-retest reliability, indicating that these two subtests may not consistently assess specific subtest functions over repeated assessments. One possible reason might be that alternate forms (blue and red forms) were used in the AF group, which may have resulted in more variation compared to using the same form as the SF group. Although the alternate forms used diminished the carryover effect, they increased the variation due to random errors in measurement. Thus, caution is needed when using the alternate forms of registration and recall subtests in people with dementia.

The MDC values can be used as thresholds to indicate whether an individual’s changed score between two successive assessments is due to real improvement or due to measurement errors [29]. For example, the MDC value of the MMSE-2:BV total score was 0.55, indicating that a change of at least 0.55 points in a successive administration of the same form of the MMSE-2:BV total score could be interpreted as a real change (i.e., beyond a random measurement error) with 95% confidence. Therefore, clinicians could use the MDC values of the MMSE-2 (with either the same or alternate forms) to interpret the change in cognitive function of an individual patient after an intervention.

Our results showed that the three versions of the MMSE-2 total scores and all subtests were all below 30% of the highest score of all test data, indicating an acceptable random measurement error. Thus, the MMSE-2 appeared reliable for describing cognitive function in people with dementia. Although the random measurement error was acceptable, we found that the MDC% of the visual construction ability subtest in both groups and the registration subtest in the AF group were higher than those in the other subtests. The large amount of MDC% values indicates that the scores of these two subtests are unstable and thus may obscure the real changes of a person with dementia. Possible reasons for these results might be the nature of practice effects and moderate test-retest reliability. Future studies are needed to investigate the causes of variations in people’s responses to visual-constructional ability and registration subtests between repeated assessments. The results could help users exclude factors that increase random measurement errors and improve the utility of the MMSE-2.

To the best of our knowledge, this was the first study to comprehensively examine the practice effect and test-retest reliability of the MMSE-2 in people with dementia. Although the number of people with severe dementia were different between the two groups (SF group = 0 vs. AF group = 9), the results of the AF group with a sample size of 51were not dramatically different when compared with the previous results using a sample size of 60 (Additional file 1). In addition, we found that all three versions of the MMSE-2 and almost all of their subtest scores, except for the story memory subtest score (*p* = 0.003), had non-significant differences at baseline (*p* > 0.05) (Additional file 2). One possible reason for the significant difference in the story memory subtest score might be due to dementia- memory problems and the discrepancy in the number of people with severe dementia in the two groups. In this case, we could expect that people with severe dementia won’t be influenced by previous testing when repeatedly assessed using the MMSE-2. In contrary to common belief that practice effect might obscure true cognitive decline, this information provides an important clue to users. Particularly, the changes in the scores of the MMSE-2 when repeatedly administered on people with severe dementia might imply that their cognitive abilities further decline [32, 33] and thus the change scores could act as an indicator to identify individuals at greater risk of clinical progression. On the basis of this implication, we believe that people with severe dementia could still gain benefit from the small practice effect of the MMSE-2. Our study results could broaden the utility of the MMSE-2, since we comprehensively examined all three versions of the MMSE-2 and proved that the MMSE-2 was useful for repeatedly screening the cognitive function of people with dementia.

Our study had five limitations. First, the participants were recruited from northern Taiwan through convenience sampling and the red forms of the MMSE-2 were not used repeatedly in our study, thus the test-retest reliability of the red forms remain unknown, which restricts the generalizability of the results in this study. Second, a two-week interval was chosen as the practice effect in this study. Different retest lengths may result in different study findings. Future studies are needed to examine the practice effect of the MMSE-2 at different time intervals. Third, the fixed order design (i.e., blue form first and red form second) used in the AF group might have influenced the results of the study. To confirm our findings, future studies that randomized the alternate form order may be needed. Fourth, the inter-rater reliability between rates has not yet been established, which may jeopardize our current validation of the MMSE-2. Future studies are needed to examine the inter-rater reliability of the MMSE-2 in people with dementia. Fifth, we did not collect information regarding whether the patients had been assessed with the MMSE or MMSE-2 before or during our study, in which case the participants might have become familiar with the screening tools. Familiarity with the screening tools might have caused underestimations of the practice effect and test-retest reliability in this study.

## Conclusion

Overall, the practice effect of the MMSE-2 diminished when the alternate forms were performed. In addition, the MMSE-2 had good to excellent test-retest reliability with an acceptable random measurement error, which supports the use of this measure in both clinical and research settings. However, the visual-constructional ability subtest showed small practice effects, and both the registration and recall subtests were found to have moderate test-retest reliability. Thus, caution should be exercised when interpreting the results of the visual-constructional ability, registration, and recall subtests of the MMSE-2.

## Supplementary Information

**Additional file 1.** Practice effects of the MMSE-2 in the alternate-form group (AF group).

**Additional file 2 **Results of independent sample *t*-test of the versions and subtests in the MMSE-2 between the SF (*n* = 60) and AF (*n* = 60) groups at baseline.

## Data Availability

Data supporting the findings are available upon request. Please contact the corresponding author, En-Chi Chiu (enchichiu@ntunhs.edu.tw), for data access.

## Abbreviations

MMSE: Mini-Mental State Examination
MMSE-2: Mini-Mental State Examination-Second Edition
MMSE-2:BV: Mini-Mental State Examination: Brief Version
MMSE-2:SV: Mini-Mental State Examination: Standard Version
MMSE-2:EV: Mini-Mental State Examination: Expanded Version
CDR: Clinical Dementia Rating

## References

[1] Alzheimer's Disease International (2015). World Alzheimer Report 2015.

[2] Sachdev PS, Blacker D, Blazer DG, Ganguli M, Jeste DV, Paulsen JS (2014). Classifying neurocognitive disorders: the DSM-5 approach. Nat Rev Neurol.

[3] Kishita N, Backhouse T, Mioshi E (2020). Nonpharmacological interventions to improve depression, anxiety, and quality of life (QoL) in people with dementia: an overview of systematic reviews. J Geriatr Psychiatry Neurol.

[4] Mograbi DC, Morris RG, Fichman HC, Faria CA, Sanchez MA, Ribeiro PC (2018). The impact of dementia, depression and awareness on activities of daily living in a sample from a middle-income country. Int J Geriatr Psychiatry.

[5] Giebel CM, Challis D, Montaldi D (2017). The newly revised interview for deteriorations in daily living activities in dementia (R-IDDD2): distinguishing initiative from performance at assessment. Int Psychogeriatr.

[6] Lee YC, Lin YT, Chiu EC (2021). A comparison of test-retest reliability of four cognitive screening tools in people with dementia. Disabil Rehabil.

[7] Folstin MF, Folstein SE, White T, Messer MA. Mini-Mental State Examination User's Manual, 2nd ed. Lutz, FL: Psychological Assessment Resources; 2010.

[8] Cao L, Ha IS, Lin X, Shu D, Wang S, Yue J, Liu G, Dong B (2012). Comparison of the saint Louis University mental status examination, the Mini-mental state examination, and the Montreal cognitive assessment in detection of cognitive impairment in Chinese elderly from geriatric department. J Am Med Dir Associ.

[9] Folstein MF, Folstein SE, McHugh PR (1975). “Mini-mental state”: a practical method for grading the cognitive state of patients for the clinician. J Psychiatr Res.

[10] Smith T, Gildeh N, Holmes C (2007). The Montreal cognitive assessment: validity and utility in a memory clinic setting. Can J Psychiatr.

[11] Song M, Lee SH, Yu KH, Kang Y (2019). Development and validation of the full version of story memory in the Korean-Mini mental state examination, 2nd edition: expanded version (K-MMSE-2: EV). Dement Neurocogn Disord.

[12] Baek MJ, Kim K, Park YH, Kim SY (2016). The validity and reliability of Mini-mental state Examination-2 for detecting mild cognitive impairment and Alzheimer’s disease in a Korean population. PLoS One.

[13] Yang H, Yim D, Park MH. Converting from the Montreal cognitive assessment to the Mini-mental state Examination-2. PLoS One. 2021; 6(7): e0254055. 10.1371/journal.pone.0254055 PMC8266092 34237113

[14] Albanna M, Yehya A, Khairi A, Dafeeah E, Elhadi A, Rezgui L (2017). Validation and cultural adaptation of the Arabic versions of the Mini-mental status examination - 2 and Mini-cog test. Neuropsychiatr Dis Treat.

[15] Mokkink LB, Terwee CB, Patrick DL, Alonso J, Stratford PW, Knol DL (2010). The COSMIN study reached international consensus on taxonomy, terminology, and definitions of measurement properties for health-related patient-reported outcomes. J Clin Eqidemiol.

[16] Chiu EC, Koh CL, Tsai CY, Lu WS, Sheu CF, Hsueh IP (2014). Practice effects and test-re-test reliability of the five digit test in patients with stroke over four serial assessments. Brain Inj.

[17] Munjir N, Othman Z, Zakaria R, Shafin N, Hussain NA, Desa AM (2015). Equivalence and practice effect of alternate forms for Malay version of auditory verbal learning test (MAVLT). EXCLI J.

[18] Yuksel E, Kalkan S, Cekmece S, Unver B, Karatosun V (2017). Assessing minimal detectable changes and test-retest reliability of the timed up and go test and the 2-minute walk test in patients with total knee arthroplasty. J Arthroplast.

[19] Lee YC, Yu WH, Hsueh IP, Chen SS, Hsieh CL (2017). Test-retest reliability and responsiveness of the Barthel index-based supplementary scales in patients with stroke. Eur J Phys Rehabil Med.

[20] Jack CR, Albert MS, Knopman DS, McKhann GM, Sperling RA, Carrillo MC, Thies B, Phelps CH (2011). Introduction to the recommendations from the National Institute on Aging-Alzheimer's Association workgroups on diagnostic guidelines for Alzheimer's disease. Alzheimers Dement.

[21] Morris JC (1991). The clinical dementia rating (CDR): current version an scoring rules. Neurology..

[22] Hughes CP, Berg L, Danziger W, Coben LA, Martin RL (1982). A new clinical scale for the staging of dementia. Br J Psychiatry.

[23] Nyunt MSZ, Chong MS, Lim WS, Lee TS, Yap P, Ng TPJD (2013). Reliability and validity of the clinical dementia rating for community-living elderly subjects without an informant. Dement Geriatr Cogn Dis Extra.

[24] Yoshinaga N, Nakamura Y, Tanoue H, MacLiam F, Aoishi K, Shiraishi Y (2018). Is modified brief assertiveness training for nurses effective? A single-group study with long-term follow-up. J Nurs Manag.

[25] Portney LG, Watkins MP (2009). Foundations of clinical research: applications to practice.

[26] Bushnell CD, Johnston DC, Goldstein LB (2001). Retrospective assessment of initial stroke severity: comparison of the NIH stroke scale and the Canadian neurological scale. Stroke..

[27] Dobson F, Hinman RS, Hall M, Marshall CJ, Sayer T, Anderson C (2017). Reliability and measurement error of the osteoarthritis research society international (OARSI) recommended performance-based tests of physical function in people with hip and knee osteoarthritis. Osteoarthr Cartil.

[28] Chiu EC, Wu WC, Chou CX, Yu MY, Hung JW (2016). Test-retest reliability and minimal detectable change of the test of visual perceptual skills-in patients with stroke.Arch Phys med. Rehabil..

[29] Huang SL, Hsieh CL, Wu RM, Tai CH, Lin CH, Lu WS (2011). Minimal detectable change of the timed “up & go” test and the dynamic gait index in people with Parkinson disease. Phys Ther.

[30] Martinelli JE, Cecato JF, Martinelli MO, de Melo BAR, Aprahamian I (2018). Performance of the pentagon drawing test for the screening of older adults with Alzheimer's dementia. Dement Neuropsychol.

[31] Mohajan HK (2017). Two criteria for good measuremetns in research: validity and reliability. Ann Spiru Haret Univ Econ Ser.

[32] Duff KB, Schultz LJ, Moser SK, McCaffrey DJ, Haase RJ, Westervelt RF, Langbehn HJ, Paulsen DR, JS. (2007). & Huntington's study group. Practice effects in the prediction of long-term cognitive outcome in three patient samples: a novel prognostic index. Arch Clin Neuropsychol.

[33] Tanaka HN, Ishimaru Y, Ogawa D, Fukuhara Y, K. Nishikawa T. (2021). Possibility of cognitive improvement in severe dementia: a case series assessed by cognitive test for severe dementia. Int J Gerontol.

