# Supplementary material for: Practice effect and test-retest reliability of the Mini-Mental State Examination-2 in people with dementia
Source: BMC Geriatr. 2022 Jan 21;22:67. doi: 10.1186/s12877-021-02732-7 (PMC8780811; doi:10.1186/s12877-021-02732-7)
Supplement: Supplementary file 1 — Additional file 1. Practice effects of the MMSE-2 in the alternate-form group (AF group). [file 12877_2021_2732_MOESM1_ESM.docx]

Additional file 1. Practice effects of MMSE-2 in the alternate-forms group (AF group)

| Versions and subtests | n=60 | | | n=51 | | |
| --- | --- | --- | --- | --- | --- | --- |
|  | paired *t*-test  (*p* value) | Cohen’s *d* | paired *t*-test  (*p* value) | | Cohen’s *d* | |
| Registration | -0.55 (0.583) | 0.06 | -0.96 (0.341) | | 0.13 | |
| Orientation | 0.07 (0.946) | 0.01 | -0.53 (0.602) | | 0.05 | |
| Recall | 0.69 (0.496) | 0.08 | 0.69 (0.497) | | | 0.09 |
| **Brief Version total score** | -0.05 (0.961) | 0.00 | -0.63 (0.533) | | | 0.06 |
| Attention and  calculation | 0.87 (0.387) | 0.07 | 0.46 (0.647) | | | 0.04 |
| Language | 0.00 (1.000) | 0.00 | -0.18 (0.856) | | | 0.02 |
| Visual‐constructional  ability | -2.65 (0.010*) | 0.26 | -2.68 (0.010*) | | | 0.32 |
| **Standard Version total score** | -0.11 (0.912) | 0.01 | -0.78 (0.441) | | | 0.06 |
| Story Memory | 0.19 (0.853) | 0.02 | 0.10 (0.854) | | | 0.02 |
| Processing Speed | -1.64 (0.106) | 0.10 | -1.65 (0.106) | | | 0.12 |
| **Expanded Version total score** | -0.75 (0.454) | 0.04 | -1.281 (0.206) | | | 0.07 |

**p*<0.05
