# Supplementary material for: Practice effect and test-retest reliability of the Mini-Mental State Examination-2 in people with dementia
Source: BMC Geriatr. 2022 Jan 21;22:67. doi: 10.1186/s12877-021-02732-7 (PMC8780811; doi:10.1186/s12877-021-02732-7)
Supplement: Supplementary file 2 — Additional file 2 Results of independent sample t-test of the versions and subtests in the MMSE-2 between the SF (n = 60) and AF (n = 60) groups at baseline. [file 12877_2021_2732_MOESM2_ESM.docx]

Additional file 2. Results of independent sample *t*-test of the versions and subtests in the MMSE-2 between the SF (n=60) and AF (n=60) groups at baseline.

| Versions and subtests | Group | Mean (SD) | *t* | *p* value |
| --- | --- | --- | --- | --- |
| Registration | SF | 2.20 (1.12) | -0.925 | 0.357 |
|  | AF | 2.00 (1.25) |  |  |
| Orientation | SF | 3.85 (2.63) | 1.292 | 0.199 |
|  | AF | 4.50 (2.88) |  |  |
| Recall | SF | 0.33 (0.71) | -0.684 | 0.495 |
|  | AF | 0.25 (0.63) |  |  |
| **Brief Version total score** | SF | 6.38 (3.59) | 0.526 | 0.600 |
|  | AF | 6.75 (4.04) |  |  |
| Attention and calculation | SF | 1.58 (1.71) | 1.117 | 0.266 |
|  | AF | 1.95 (1.88) |  |  |
| Language | AF | 5.33 (1.61) | -0.854 | 0.395 |
|  | AF | 5.03 (2.19) |  |  |
| Visual‐constructional ability | SF | 0.40 (0.49) | 0.184 | 0.854 |
|  | AF | 0.42 (0.50) |  |  |
| **Standard Version total score** | SF | 13.70 (5.74) | 0.366 | 0.715 |
|  | AF | 6.75 (4.04) |  |  |
| Story Memory | SF | 3.22 (2.60) | -3.026 | 0.003^*^ |
|  | AF | 1.77 (2.65) |  |  |
| Processing Speed | SF | 3.87 (4.07) | -0.557 | 0.579 |
|  | AF | 3.45 (4.14) |  |  |
| **Expanded Version total score** | SF | 20.78 (10.53) | -0.688 | 0.493 |
|  | AF | 19.35 (12.22) |  |  |

SF group: Same-forms group; AF group: alternate-forms group

**p*<0.05
